# Uric acid: association with rate of renal function decline and time until start of dialysis in incident pre-dialysis patients

**DOI:** 10.1186/1471-2369-15-91

**Published:** 2014-06-16

**Authors:** Hakan Nacak, Merel van Diepen, Moniek CM de Goeij, Joris I Rotmans, Friedo W Dekker

**Affiliations:** 1 Department of Clinical Epidemiology, Leiden University Medical Center, Albinusdreef 2, Leiden 2333 ZA, The Netherlands; 2 Department of Nephrology, Leiden University Medical Center, Leiden, The Netherlands

**Keywords:** Uric acid, CKD progression, Pre-dialysis, Prospective cohort

## Abstract

**Background:**

In patients with chronic kidney disease (CKD) hyperuricemia is common. Evidence that hyperuricemia might also play a causal role in vascular disease, hypertension and progression of CKD is accumulating. Therefore, we studied the association between baseline uric acid (UA) levels and the rate of decline in renal function and time until start of dialysis in pre-dialysis patients.

**Methods:**

Data from the PREPARE-2 study were used. The PREPARE-2 study is an observational prospective cohort study including incident pre-dialysis patients with CKD stages IV-V in the years between 2004 and 2011. Patients were followed for a median of 14.9 months until start of dialysis, kidney transplantation, death, or censoring. Main outcomes were the change in the rate of decline in renal function (measured as estimated glomerular filtration rate (eGFR)) estimated using linear mixed models, and time until start of dialysis estimated using Cox proportional hazards models.

**Results:**

In this analysis 131 patients were included with a baseline UA level (mean (standard deviation (SD)) of 8.0 (1.79) mg/dl) and a mean decline in renal function of -1.61 (95% confidence interval (CI), -2.01; -1.22) ml/min/1.73 m^2^/year. The change in decline in GFR associated with a unit increase in UA at baseline was -0.14 (95% CI -0.61;0.33, p = 0.55) ml/min/1.73 m^2^/year. Adjusted for demography, comorbidities, diet, body mass index (BMI), blood pressure, lipids, proteinuria, diuretic and/or allopurinol usage the change in decline in eGFR did not change. The hazard ratio (HR) for starting dialysis for each mg/dl increase in UA at baseline was 1.08 (95% CI, 0.94;1.24, p = 0.27). After adjustment for the same confounders the HR became significant at 1.26 (95% CI, 1.06;1.49, p = 0.01), indicating an earlier start of dialysis with higher levels of UA.

**Conclusion:**

Although high UA levels are not associated with an accelerated decline in renal function, a high serum UA level in incident pre-dialysis patient is a risk factor for an earlier start of dialysis.

## Background

Uric acid (UA) is an emerging risk factor for renal disease, hypertension, and cardiovascular disease. Hyperuricemia is common in patients with chronic kidney disease (CKD) and evidence that hyperuricemia may also play a causal role in hypertension, vascular disease and progression of CKD is accumulating [1-9]. In addition, some intervention studies have shown that treatment of hyperuricemia could be beneficial for blood pressure regulation and preservation of kidney function [10,11]. Therefore, screening for hyperuricemia in CKD patients might help to identify patients that have an accelerated decline in renal function and thereby an increased risk for progression to ESRD.

The association between UA and decline in renal function has been investigated in several studies which included healthy individuals [12,13], patients with CKD stages I-II [14,15], patients with diabetes [6], and patients on peritoneal dialysis [16]. However, evidence about this association in patients with advanced CKD (stages IV-V) is scarce, and the effect on time until start of dialysis has not been addressed specifically.

The aim of our study is to investigate the association between baseline UA levels and the annual rate of decline in renal function and time until start of dialysis in CKD stage IV-V patients referred to specialized pre-dialysis care. We hypothesize that UA in these patients is associated with accelerated decline in renal function and an earlier start of dialysis.

## Methods

### Study design

The association between UA and the annual rate of decline in renal function and time until start of dialysis was investigated in the ongoing PRE-dialysis PAtient REcord-2 (PREPARE-2) study, an observational prospective cohort study in incident pre-dialysis care patients. These patients were recruited from 25 nephrology outpatient clinics in the Netherlands. They were regularly seen by their nephrologist and treated in accordance with the treatment guidelines of the Dutch Federation of Nephrology, which are partly based on the K/DOQI and EBPG guidelines [17-20]. Data were collected in 6-monthly intervals from the start of pre-dialysis care onwards. Patients were followed until start of dialysis, kidney transplantation, death or censoring. Patients were censored if they refused to further participate, if they moved to an outpatient clinic not participating in the PREPARE-2 study, if they were lost to follow-up, if their kidney function normalized, or if their follow-up was still continuing on February 12, 2013, whichever came first. The medical ethics committee or institutional review boards (when appropriate) of all participating centers approved this study (see Additional file 1: ‘Ethical approval Prepare-2 study’).

### Study population

Patients were eligible for inclusion if they were 18 years or older and had been referred to a specialized pre-dialysis outpatient clinic. In practice, this meant that the included pre-dialysis patients had an estimated glomerular filtration rate (eGFR) of less than 20–30 ml/min/1.73 m^2^ accompanied by a progressive decline in renal function. Patients were also included if they had a failing transplant and had been transplanted at least one year ago. Prior to inclusion all patients gave written informed consent.

### Measurement and definitions

In addition to the routine collection, laboratory data were also extracted from the electronic hospital information systems or medical records. The closest laboratory measurement performed within 90 days before or after the date of a visit was appointed to that visit. When no serum UA measurement was available on the first visit, but it was measured during the second visit, then this value was defined as the baseline value for UA. Variables used in the multivariable analysis were determined on the same visit as UA. Patients were regarded as hypertensive if a physician had diagnosed them as such. Proteinuria was defined as >300 mg/day protein in urine. The eGFR was calculated using the 4-variable Modification of Diet in Renal Disease (MDRD) formula, taking into account age, sex, black race, and serum creatinine [21].

### Outcomes

The main outcomes are the change in rate of decline in renal function and time until start of dialysis per mg/dl increase in baseline UA. To calculate the rate of decline in renal function, all available eGFR measurements from three months prior to inclusion until end of follow-up were used. In patients initiating dialysis, eGFR measurements until two weeks before the start of dialysis were used, because eGFR measurements after this point in time were no longer representative of true kidney function. Start of dialysis was defined as starting HD or PD during follow-up.

### Statistical analysis

Continuous variables are presented as mean ± standard deviation (SD), skewed variables are presented as median (boundaries of interquartile range, IQR) and categorical variables are presented as percentages. Baseline characteristics are presented for the total study population and stratified by patients below or above the median serum UA level at baseline.

A linear mixed model (LMM) was used to estimate the change in the rate of decline in renal function with each mg/dl higher UA level at baseline [22]. In contrast to a standard linear model, the LMM takes into account that repeated eGFR measurements of the same patient are correlated. Multivariable analysis was used to adjust for potential confounders and we defined two adjusted models: age, sex, primary kidney disease, body mass index (BMI), presence of cardiovascular disease (CVD; angina pectoris, coronary disease, history of cerebrovascular accidents, heart failure, and/or myocardial infarction), ethnicity, hypertension, presence of diabetes mellitus (DM; type 1 and 2), protein restricted diet, systolic blood pressure (model 1) and model 1 plus low-density lipoprotein (LDL), cholesterol, proteinuria, diuretic use and allopurinol use (model 2) .

Kaplan-Meier analysis was performed to produce survival plots [23]. A Cox proportional hazards analysis was used to estimate the hazard ratio (HR) for starting dialysis with each mg/dl higher UA level at baseline [24]. Follow-up time in this Cox proportional hazards analysis was the time between the baseline UA measurement and the start of dialysis. Mortality, kidney transplantation, loss to follow up, refusal of further participation, transfer to another outpatient clinic that doesn’t participate in the PREPARE-2 study and follow-up continuing until at least February 12, 2013 were treated as censored events. The multivariable Cox analysis was adjusted for the same confounders as the LMM.

Missing values of potential confounders at baseline were imputed for patients with a baseline UA value using multiple imputation with 10 repetitions. The imputed data are predicted based on known information of each individual [25]. Besides the variables in model 1 and 2, diastolic blood pressure, high density lipoprotein, triglycerides, baseline eGFR, follow-up time and the endpoint reached (start of dialysis or not) were used for imputation. Follow-up time was skewed and was therefore logarithmically transformed before entering into the model.

Multiple sensitivity analyses were performed to test the robustness of our findings. First, we repeated our two main analyses without imputing missing confounder data. Second, alongside the main analysis (i.e. UA on a continuous scale) we dichotomized UA levels based on the normal values for UA (i.e. 7.06 mg/dl for men and 5.72 for women). Third, we added baseline eGFR levels to our models, in order to adjust for potential discrepancies in the eGFR at baseline.

P < 0.05 was considered statistically significant. All analyses were performed in PASW/SPSS version 20.0 for Windows.

## Results

### Patient characteristics

In total, 502 incident pre-dialysis patients were included in the PREPARE-2 study and prospectively followed. A total of 131 patients had a baseline UA measurement. Patients with a UA measurement at the first visit (0 months) did not differ from patients with a UA measurement at the second visit (6 months) (data not shown). Baseline characteristics for the total population and for patients with UA levels above and below the median are shown in Table 1. The 131 patients had a median UA level of 7.9 mg/dl at baseline, mean (SD) age was 63.6 (14.6) years and 68.7% of the patients were male. If UA levels at baseline were above the median UA level, patients were more often male (75.4% versus 62.9%). They were also more often hypertensive (83.6% vs. 74.3%), had less often proteinuria (66.7% vs. 85.7%), and used more often diuretics (55.7% vs. 44.1%). Allopurinol was used less often in patients with UA levels above the median (4.9% vs. 29.4%). Of the 19 variables that were used to impute missing data 9 were complete and 6 variables had less than 5% missing data. On average the percentage of missing data per confounder was 7.7%.

**Table 1:** Baseline characteristics for patients with UA levels above and below median and all patients

	**UA ≤ 7.9 mg/dl**	**UA > 7.9 mg/dl**	**Total**
	**(median)**	**(median)**	**(n = 131)**
	**(n = 70)**	**(n = 61)**	
UA (mg/dl) (*sd*)	6.69 (0.89)	9.5 (1.32)	8.0 (1.79)
Age (years) *(sd)*	64.0 (14.0)	63.2 (15.4)	63.6 (14.6)
Sex (*% male*)	62.9	75.4	68.7
Ethnicity (*% caucasian*)	94.3	98.4	96.2
Diabetes *(%)*	20.0	19.7	19.8
PKD			
% *Diabetes*	7.1	9.8	8.4
% *Glomerulonephritis*	12.9	13.1	13.0
% *Renal vascular disease*	27.1	34.4	30.5
% *Other*	52.9	42.6	48.1
Hypertension* *(%)*	74.3	83.6	78.6
BMI (kg/m^2^) *(sd)*	26.3 (4.8)	25.0 (3.9)	25.7 (4.4)
CVD *(%)*	35.7	44.3	39.7
eGFR (MDRD)** *(sd)*	16.9 (5.7)	16.4 (5.7)	16.8 (5.7)
SBP (mm Hg) *(sd)*	143.9 (21.6)	141.4 (17.9)	142.7 (19.9)
DBP (mm Hg) *(sd)*	78.8 (12.3)	78.7 (10.9)	78.8 (11.6)
Total cholesterol (mmol/L) *(sd)*	4.33 (0.95)	4.48 (1.0)	4.42 (0.98)
LDL (mmol/L) *(sd)*	2.37 (0.78)	2.52 (0.95)	2.47 (0.87)
Proteinuria*** *(%)*	85.7	66.7	67.7
Diuretics *(%)*	44.1	55.7	50.4
Protein-restricted diet *(%)*	73.1	80.3	76.9
Allopurinol *(%)*	29.4	4.9	18.2

PKD = Primary kidney disease, BMI = Body Mass Index, CVD = Cardiovascular disease, SBP = systolic blood pressure, DBP = diastolic blood pressure, LDL = low-density lipoprotein.

*As diagnosed by a physician.

**in ml/min/1.73 m^3^.

***defined as >300 mg/day in urine.

### Decline in renal function

The patients had on average 3.46 (1.80) measurements of eGFR during follow-up. For 125 patients an eGFR measurement was available at baseline and the mean (SD) eGFR for these patients was 16.6 (5.7) ml/min/1.73 m^2^. The mean decline in renal function was -1.61 ml/min/1.73 m^2^/year (95% CI -2.01, -1.22); indicating a loss in renal function over time.

Each mg/dl higher UA level at baseline was associated with a change in the rate of decline in renal function of -0.14 ml/min/1.73 m^2^/year (95% CI -0.61,0.33). After adjustment for age, sex, ethnicity, primary kidney disease (PKD), comorbidities (CVD, DM, hypertension), BMI, protein restricted diet, systolic blood pressure (model 1) each mg/dl higher UA level at baseline was associated with a change in the rate of decline in renal function of -0.05 ml/min/1.73 m^2^/year (95% CI -0.56,0.47). Additional adjustment for LDL, cholesterol, proteinuria, diuretic use, and allopurinol use (model 2) again resulted in an associated change in decline in renal function of -0.14 ml/min/1.73 m^2^/year (95% CI -0.70,0.42) per mg/dl UA (Table 2).

**Table 2:** Linear mixed model for the annual rate of decline in renal function

**Mean decline in eGFR (ml/min/1.73 m**^**2**^**)**	**-1.61 (95% CI -2.01; -1.22)**
**Change in decline in eGFR per mg/dl increase in UA at baseline**	
Crude (n = 129)	-0.14 (95% CI -0.61; 0.33)
Adjusted for Model 1 (n = 129)	-0.05 (95% CI -0.56; 0.47)
Adjusted for Model 2 (n = 129)	-0.14 (95% CI -0.70; 0.42)

Model 1 = Age, sex, ethnicity, PKD, BMI, CVD, hypertension, DM, protein restricted diet, SBD.

Model 2 = Model 1 + LDL, cholesterol, proteinuria, diuretics, allopurinol.

### Time until start of dialysis

**Figure 1:**
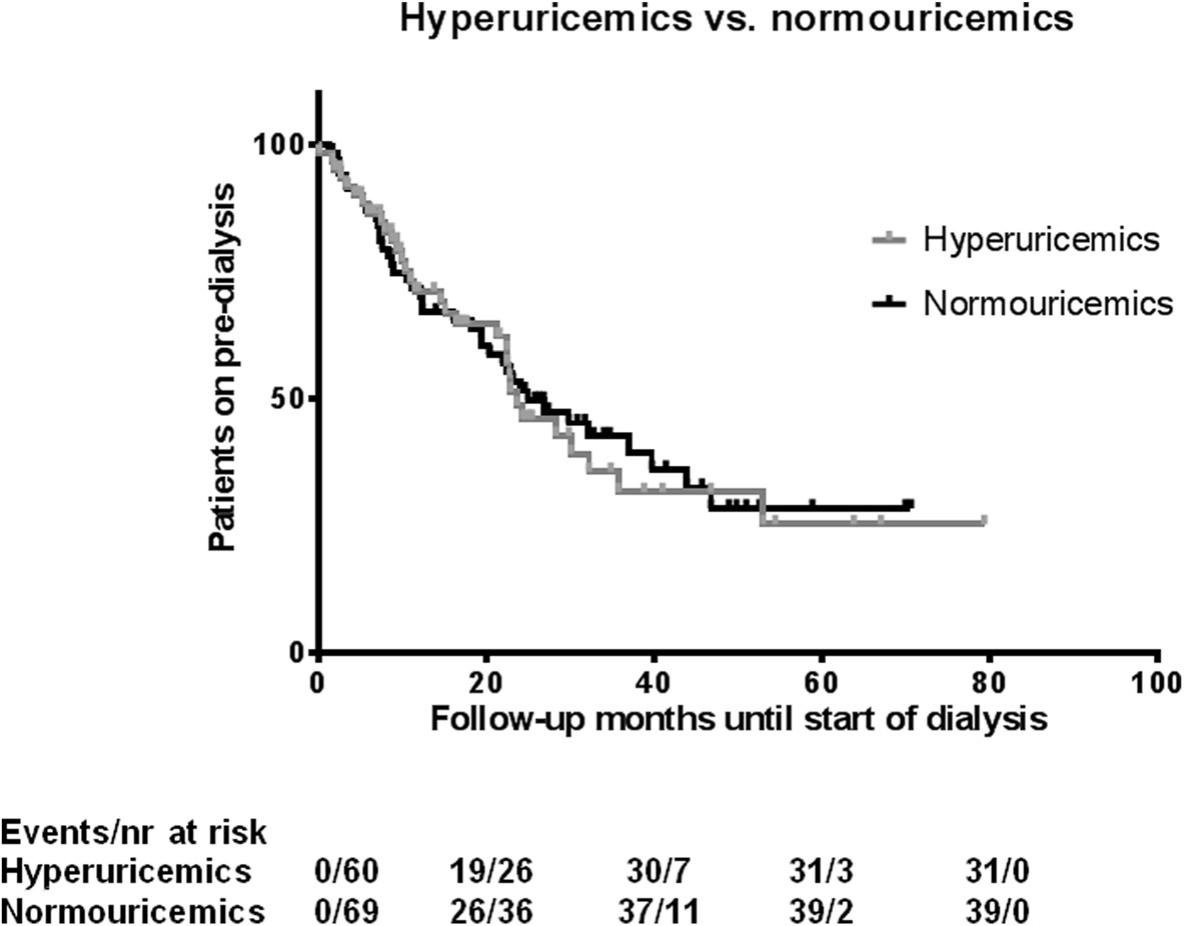
Kaplan-Meier for start of dialysis between hyperuricemics versus normouricemics.

Seventy-one (54.2%) patients started dialysis during follow up. Hemodialysis was modality of choice for 40 (56.3%) patients, the remaining 31 (43.7%) patients started with peritoneal dialysis. During pre-dialysis care 9 (6.8%) patients died. Eighteen (13.7%) patients were lost to follow-up; 9 (50.0%) of which had a UA level above the median level. Thirteen (9.9%) patients were still on pre-dialysis care at the end of follow up (February 12, 2013). Median follow-up time was 16.4 (IQR 7.7-25.4) and 20.4 (IQR 7.6-32.3) months for patients with UA levels above the median and below the median level, respectively. Figure 1 shows the Kaplan-Meier curves for those two patient groups. Patients showed a higher probability for starting dialysis if UA levels were above the median level at baseline.

The crude Cox proportional hazards analysis resulted in a HR for starting dialysis of 1.08 (95% CI 0.94 – 1.24; p = 0.27) for each mg/dl higher UA level at baseline. Adjustment for variables in model 1 resulted in a HR for starting dialysis of 1.18 (95% CI 1.01 – 1.38; p = 0.041). Additional adjustment for variables in model 2 increased the HR for starting dialysis to 1.26 (95% CI 1.06 – 1.49; p = 0.009) (Table 3).

**Table 3:** Cox proportional hazards model for time until start of dialysis

**UA per mg/dl increase at baseline**	
Crude HR (n = 131)	1.08 (95% CI 0.94; 1.24)
Adjusted HR model 1 (n = 131)	1.18 (95% CI 1.00; 1.38)
Adjusted HR model 2 (n = 131)	1.26 (95% CI 1.06; 1.50)

Model 1 = Age, sex, ethnicity, PKD, BMI, CVD, hypertension, DM, protein restricted diet, SBD.

Model 2 = Model 1 + LDL, cholesterol, proteinuria, diuretics, allopurinol.

### Sensitivity analyses

Our sensitivity analyses showed robustness of results. First, results for LMM and Cox proportional hazards analyses without imputation of missing data were similar to and in line with the results based on the imputed data. Second, we dichotomized based on normal UA levels in men and women. Results did not change for different UA categories in any of the analyses. Third, adding baseline eGFR to the multivariable models did not substantially change results.

## Discussion

Our study investigated the rate of decline in renal function and time until start of dialysis associated with each mg/dl higher UA level. Crude analyses showed that each mg/dl higher UA level was associated with a 1.08-fold higher rate of starting dialysis. After extensive adjustment for potential confounding the HR increased to 1.26. Our analyses showed that UA levels at baseline were not associated with the rate of decline in renal function.

### Uric acid and decline in renal function

Previous animal studies have shown that UA can lead to glomerular hypertension, elevated renal vascular resistance, reduced renal blood flow [26-28], activation of renin-angiotensin system (RAS), arteriolosclerosis, glomerular hypertrophy, glomerulosclerosis, and interstitial disease [29,30] by inducing oxidative stress and endothelial dysfunction indicating that UA could contribute to renal damage.

Several studies have been conducted on the relation between baseline UA levels and the decline in renal function, but data in patients with CKD stage IV-V are limited. Kuo et al. studied the association between hyperuricemia and annual decline in eGFR in 63,758 patients with an initial eGFR ≥ 60 ml/min/1.73 m^2^ and no gout in Taiwan [14]. The patients with hyperuricemia (n = 11,869) had an annual decline of eGFR that was almost twice as high as patients with normouricemia, 2.5 vs. 1.3 ml/min/1.73 m^2^. Bellomo et al. followed 824 healthy people for 5 years and found that UA is an independent risk factor for GFR decrease [12]. Zhang et al. also investigated the association between UA levels and GFR decline in 1,410 healthy participants with baseline eGFR > 60 ml/min/1.73 m^2^ in Beijing, China. Results showed that each mg/dl increase at baseline is associated with 19% more risk for GFR decline [15]. Obermayr et al. followed 21,475 healthy volunteers for 7 years in order to examine the association between hyperuricemia and the development of new-onset kidney disease, which was defined as eGFR < 60 ml/min/1.73 m^2^. After adjustment, patients in the third tertile (UA ≥ 9.0 mg/dl) had a 2.49 times higher risk of developing CKD (stage III; eGFR < 60 ml/min/1.73 m^2^) [13]. Weiner et al. performed a similar study in 13,338 participants of the Atherosclerosis Risks in Communities study or the Cardiovascular Health Study. Those patients were followed for an average of 8.5 years and had intact renal function. Adjusted results showed that each mg/dl increase in UA level led to a 1.07 times higher risk of getting CKD (stage III; eGFR < 60 ml/min/1.73 m^2^) [31]. Sturm et al. studied the association between UA levels and CKD progression in 177 non-diabetic CKD patients (stage I-V) that were followed for seven years. They found that UA was not predictive for CKD progression. However, in analyses excluding patients using UA lowering drugs all results pointed towards a faster progression in patients with high levels of UA [32]. In the MDRD study the associations between UA at baseline and all-cause mortality, CVD mortality and kidney failure were studied in CKD stages III–IV patients. In this study tertiles of UA were not associated with kidney failure [33].

Except for the last two studies, the patients included were healthier or had less severe CKD compared with the patients in our study. These studies demonstrated an association between UA at baseline and the rate of decline in renal function, whereas this association was not present in the last two studies and our study. This suggests that the effects of UA levels at baseline on the rate of decline in renal function differ between CKD stages.

### Uric acid and start of dialysis

High UA levels at baseline were associated with a shorter time until start of dialysis. Since UA was not associated with decline in renal function in our cohort, this association might be explained by clinical symptoms such as gout that relate to UA accumulation. Gout is caused by deposition of UA crystals in the joints, most notably in the metatarsal-interphalangeal joint of the big toe. Painful and disabling symptoms of gout arthritis could have contributed to the decision of the nephrologist and patient to start dialysis. Furthermore, another explanation could be that high levels of UA might have resulted in other symptoms or clinical conditions such as renal stones or hypertension that affect the decision to start dialysis.

### Strengths and limitations

A major strength of the PREPARE-2 study is the prospective longitudinal design in which the course of renal function can be investigated. However, it has been estimated that 36-65% of people in the general population with an eGFR < 15 ml/min/1.73 m^2^ are not treated by a nephrologist [34]. While this might hamper the generalizability of our results to all patients with an eGFR < 15 ml/min/1.73 m^2^, our findings can readily be generalized to the clinical practice of pre-dialysis care. As the majority of our cohort was Caucasian (94%), our results may be different in Afro-American, Asian, and other populations.

Our analyses focused on a subset of 131 patients with a baseline UA measurement. In theory, it is possible that these 131 patients, which might be considered a limited size sample, form a selected subgroup. However, we believe the availability of serum UA levels is independent of a patient’s rate of decline in renal function or expected time until start of dialysis. Therefore, we believe that our 131 patients form a random sample of incident patients on pre-dialysis care. Some patients had missing data for laboratory measurements. We used the method of multiple imputation to impute these missing values for each patient, further reducing bias [25].

The PREPARE-2 study is an observational cohort study, which means that it is possible that patients with increased UA levels are treated differently because their physician feels differently about their prognosis. However, as the effect of UA on the rate of decline in renal function and time until start of dialysis in this population has not been studied before, this is unlikely to have caused bias. Moreover, in our analyses we adjusted for known risk factors for an accelerated decline in renal function and early start of dialysis, further reducing potential confounding. While other differences in treatment policies, for instance earlier start of dialysis in more recent years might have occurred, it is most likely that these were present equally in patients with high versus low UA levels.

Not unexpectedly, we found patients treated with allopurinol had lower UA levels. As we assume allopurinol had no direct or pleiotropic effects on the rate of decline in renal function or time until start dialysis, use of allopurinol is not considered a potential confounder. This was confirmed in our analysis, as removing allopurinol from model 2 showed effects which were not materially different (data not shown). Moreover, allopurinol is prescribed as preventive treatment in patients with recurrent gout, which means that physicians are unlikely to more easily prescribe allopurinol to patients that they feel are at risk to start early.

## Conclusion

Higher UA levels in incident pre-dialysis patients are a risk factor for an early start of dialysis, although no association with accelerated decline in renal function was found. This may indicate that patients with higher UA levels should be referred earlier to pre-dialysis care in order to guarantee appropriate preparation for start of dialysis. In the future UA levels might guide nephrologists in assessing the optimal moment to start dialysis, because we have established that higher baseline UA levels lead to an earlier start of dialysis, independent of other factors.

Therefore, more research is needed that focuses on the association between individual patient signs, symptoms, laboratory values and survival and quality of life on dialysis. This association will be investigated in the European EQUAL study that can aid nephrologists and patients in making a more evidence based decision regarding the need to start dialysis for a specific patient. In the EQUAL study UA data will be collected.

## Competing interests

The authors have had no involvements that might raise the question of bias in the work reported or in the conclusions, implications, or opinions stated. None of the sponsors were involved in study design, collection of data, statistical analyses, interpretation of data, writing of the manuscript, or in the decision to submit the paper for publication. The results presented in this paper have not been published previously in whole or part, except in abstract format.

## Authors’ contributions

HN performed the statistical analyses and drafted the manuscript. MvD, MCMdG, JI, and FWD contributed to the conception and the design and revised the manuscript critically. All authors have given final approval for this version to be submitted.

## Pre-publication history

The pre-publication history for this paper can be accessed here:

http://www.biomedcentral.com/1471-2369/15/91/prepub

## Supplementary Material

Additional file 1 Ethical approval Prepare-2 study. Click here for file

## The PREPARE-2 Study Group consists of

I. Keur and R.T. Krediet, Amsterdam Medical Center, Amsterdam;

H. Brulez, St Lucas Andreas Hospital, Amsterdam;

C.E. Douma, VU Medical Center, Amsterdam;

Y. Vermeeren and J. Barendregt, Gelre Hospital, Apeldoorn;

R. Bakker, Amphia Hospital, Breda;

H. Boom, Reinier de Graaf Gasthuis, Delft;

E. Hoogeveen, Jeroen Bosch Hospital, Den Bosch;

M. Groeneveld, Haga Hospital, The Hague;

M. den Hartog and G.W. Feith, Gelderse Vallei Hospital, Ede;

S. Konings, Catharina Hospital, Eindhoven;

G.H. Vastenburg, Scheper Hospital, Emmen;

A. Lavrijsen,Oosterschelde Hospital, Goes;

J.W. Eijgenraam, Groene Hart Hospital, Gouda;

C.F.M. Franssen, University Medical Center Groningen, Groningen;

C.A. Verburgh, Kennemer Gasthuis, Haarlem;

M.H. Hemmelder, Medical Center Leeuwarden, Leeuwarden;

Y.W.J. Sijpkens, Leiden University Medical Center, Leiden;

A. Schrander-van der Meer, Rijnland Hospital, Leiderdorp;

S. Boorsma, Laurentius Hospital, Roermond;

T. Noordzij and H. Krepel, Franciscus Hospital,Roosendaal;

Y. Schrama, St. Franciscus Gasthuis, Rotterdam;

C. Beerenhout and J. van Ampting, Maxima Medical Center, Veldhoven;

M. Ho-dac-Pannenkeet, Zaans Medical Center, Zaandam;

S.H.A. Diepeveen, Isala Klinieken, Zwolle - all in The Netherlands.

D.C. Grootendorst, Leiden University Medical Center, Leiden

M. Verduijn, Leiden University Medical Center, Leiden

M.C. de Goeij, Leiden University Medical Center, Leiden

D.J. de Jager, Leiden University Medical Center, Leiden

E.W. Boeschoten, Hans Mak Institute, Naarden, Leiden

F.W. Dekker, Leiden University Medical Center, Leiden
