# Supplementary material for: Uric acid: association with rate of renal function decline and time until start of dialysis in incident pre-dialysis patients
Source: BMC Nephrol. 2014 Jun 16;15:91. doi: 10.1186/1471-2369-15-91 (PMC4075499; doi:10.1186/1471-2369-15-91)
Supplement: Additional file 1 — Ethical approval Prepare-2 study. [file 1471-2369-15-91-S1.doc]

**Additional file: Ethical approval PREPARE-2 Study**

**Uric acid: association with rate of renal function decline and time until start of dialysis in incident pre-dialysis patients**

The research protocol of the PREPARE-2 study was evaluated and approved by the medical ethics committee of the Leiden University Medical Center (LUMC). According to Dutch law the medical ethics committee or institutional review board (as appropriate) of the other participating centers only evaluated the local feasibility of the study and thereafter gave their approval. These centers are:

- Amsterdam, Academic Medical Center
- Amsterdam, Sint Lucas-Andreas Hospital
- Amsterdam, VU Medical Center
- Apeldoorn, Gelre Hospitals
- Breda, Amphia Hospital
- Delft, Reinier de Graaf
- Den Bosch, Jeroen Bosch Hospital
- The Hague: Medical Center Haaglanden
- Ede, Hospital Gelderse Vallei
- Eindhoven, Catharina Hospital
- Emmen, Scheper Hospital (approval also included Beilen, Dialysis Clinic North)
- Goes, Admiraal de Ruyter Hospital
- Gouda, Groene Hart Hospital
- Groningen, University Medical Center Groningen
- Haarlem, Kennemer Gasthuis
- Leeuwarden, Medical Center Leeuwarden
- Leiden, Leiden University Medical Center
- Leiderdorp, Rijnland Hospital
- Roermond, Laurentius Hospital
- Roosendaal, Franciscus Hospital
- Rotterdam, Franciscus Gasthuis
- Veldhoven, Máxima Medical Center
- Zaandam, Zaans Medical Center
- Zwolle, Isala Clinics
